# Steroids in fluid and/or vasoactive infusion dependent pediatric shock: study protocol for a randomized controlled trial

**DOI:** 10.1186/s13063-016-1365-6

**Published:** 2016-05-06

**Authors:** Katharine O’Hearn, Dayre McNally, Karen Choong, Anand Acharya, Hector R. Wong, Margaret Lawson, Tim Ramsay, Lauralyn McIntyre, Elaine Gilfoyle, Marisa Tucci, David Wensley, Ronald Gottesman, Gavin Morrison, Kusum Menon, On behalf of the Canadian Critical Care Trials Group

**Affiliations:** Research Institute, Children’s Hospital of Eastern Ontario, Ottawa, Canada; Department of Pediatrics, Faculty of Medicine, University of Ottawa, Children’s Hospital of Eastern Ontario, Ottawa, Canada; McMaster Children’s Hospital, McMaster University, Hamilton, Canada; Department of Economics, Faculty of Public Affairs, Carleton University, Ottawa, Canada; Cincinnati Children’s Hospital Medical Center, University of Cincinnati College of Medicine, Cincinnati, USA; Department of Epidemiology, University of Ottawa and Ottawa Hospital Research Institute (OHRI), University of Ottawa, Ottawa, Canada; Clinical Epidemiology Program, The Ottawa Hospital Research Institute (OHRI), Ottawa, Canada; Department of Medicine (Division of Critical Care), Ottawa Hospital Research Institute (OHRI), University of Ottawa, Ottawa, Canada; Section of Critical Care Medicine, Department of Pediatrics, Alberta Children’s Hospital, Calgary, Canada; Department of Pediatrics, CHU Sainte-Justine Hospital, Montreal, Canada; Department of Pediatrics, Faculty of Medicine, The University of British Columbia, British Columbia Children’s Hospital, Vancouver, Canada; Department of Pediatrics, Faculty of Medicine, McGill University, Montreal Children’s Hospital, Montreal, Canada; Department of Critical Care Medicine, IWK Health Centre, Halifax, Canada

## Abstract

**Background:**

Physicians often administer corticosteroids for the treatment of fluid and vasoactive infusion dependent pediatric shock. This use of corticosteroids is controversial, however, and has never been studied in a pediatric randomized controlled trial (RCT). This pilot trial will determine the feasibility of a larger RCT on the role of corticosteroids in pediatric shock.

**Methods/design:**

Steroids in Fluid and/or Vasoactive Infusion Dependent Pediatric Shock (STRIPES) is a pragmatic, seven-center, double-blind, pilot RCT. We aim to randomize 72 pediatric patients with fluid and vasoactive infusion dependent shock to receive either hydrocortisone or a saline placebo for 7 days or until clinical stability, whichever occurs first. The primary outcome of this pilot trial is the feasibility of recruitment, defined as the number of patients enrolled over a 1-year period. Secondary outcomes include the frequency of, and reasons for, open-label steroid use, protocol adherence, incidence of mortality and corticosteroid-associated adverse events, time to discontinuation of inotropes, and feasibility of blood sampling.

**Discussion:**

Corticosteroids are used for the treatment of pediatric shock without sufficient evidence to support this practice. While there is a scientific rationale and limited data supporting their use in this setting, there is also evidence from other populations suggesting potential harm. The STRIPES pilot study will assess the feasibility of a larger, much needed trial powered for clinically important outcomes.

**Trial registration:**

ClinicalTrials.gov: NCT02044159

**Electronic supplementary material:**

The online version of this article (doi:10.1186/s13063-016-1365-6) contains supplementary material, which is available to authorized users.

## Background

Fluid and vasoactive infusion dependent shock is a critical form of cardiovascular failure affecting approximately 20,000 North American children annually. This form of severe shock results in approximately 5 % of pediatric intensive care unit admissions [1] and may lead to multi-organ failure with significant morbidity [2, 3] and a 2–10 % mortality rate [4, 5]. The role of corticosteroids in pediatric patients with severe septic shock who are not responding to fluids and vasoactive infusions has been widely debated for more than 40 years, and current clinical research evidence neither supports nor refutes this practice [6, 7].

The American College of Critical Care Medicine states that “fluid and vasoactive infusion dependent shock results from inadequate cellular corticosteroid activity for the severity of the patient’s illness” [8]. This condition has been referred to as “relative adrenal insufficiency” or “critical illness related adrenal insufficiency” [9, 10] and is a complex condition resulting from a variety of mechanisms, making it challenging to define and diagnose [10, 11]. Lack of sufficient cortisol leads to hemodynamic instability through decreased myocardial contractility, increased vasodilatation, and/or capillary leak syndrome [12, 13]. Thus, there is some scientific rationale for corticosteroid use in this population; however, the small numbers of observational studies and randomized controlled trials on this subject have reported conflicting conclusions.

Eight small randomized controlled trials (RCTs) of corticosteroids in pediatric shock have included a total of 489 patients [2, 3, 14–19]. Each study enrolled fewer than 100 patients and had methodological limitations, and all were conducted in developing nations with a focus on dengue shock. Two studies demonstrated a mortality benefit from corticosteroids in dengue shock syndrome [14, 18], and the remaining trials were not adequately powered to determine the effect of corticosteroids on clinically relevant outcomes. None of the trials were conducted since the publication of the Surviving Sepsis Campaign guidelines [20], making them difficult to interpret in the context of current shock management. Additionally, heterogeneity in steroid agents, dosing regimens, and duration of therapy make interstudy comparisons difficult. Similarly, the two largest RCTs conducted involving critically ill adults also demonstrated contradictory results [21, 22].

The potential adverse effects of corticosteroids in pediatric shock have not been rigorously evaluated. Recent observational studies suggest that corticosteroid therapy in this population may increase hyperglycemia and secondary infections, suppress adaptive immunity, and increase mortality [23–25]. An adult RCT also suggested an increase in secondary infections and mortality with the use of corticosteroids in shock [21].

Given the poor methodological quality of the existing pediatric RCTs, the variability of administration protocols studied, contradictory results from adult trials, and the lack of information on potential adverse effects, it has been difficult to develop evidence-based guidelines for steroid use in critically ill children with fluid and vasoactive infusion dependent shock. Hydrocortisone is the most commonly used corticosteroid in large randomized controlled shock trials [21, 22] and is currently the most commonly used corticosteroid for treatment of pediatric shock [26]. However, the management of these patients remains highly variable with many critical care physicians having strongly held beliefs both for and against steroid use [27, 28].

We therefore propose a pragmatic, parallel group, randomized, placebo-controlled pilot trial of hydrocortisone for the treatment of pediatric fluid and vasoactive infusion dependent shock. The specific objectives of the STRIPES (Steroids in Fluid and Vasoactive Infusion Dependent Pediatric Shock) pilot study are to estimate the rate of patient recruitment and understand barriers to recruitment, assess the appropriateness of our eligibility criteria for a full trial, assess adherence to the treatment protocol, and assess the feasibility of collecting and shipping blood samples.

## Methods/design

This protocol adheres to the Standard Protocol Items: Recommendations for Interventional Trials (SPIRIT) checklist (see checklist, Additional file 1). A summary of the protocol is provided in Table 1.

**Table 1:** World Health Organization trial registration data set: structured summary

Data category	Information
Primary registry, trial identifying number	ClinicalTrials.gov identifier NCT02044159
Date of registration in primary registry	January 21, 2014
Secondary identifying numbers	CHEO REB 14/05E
Protocol version	Version 5, May 7, 2015
Sources of monetary support	Canadian Institutes of Health Research Operating Grant
Primary sponsor	Investigator-initiated study
	Kusum Menon (KM)
	Children’s Hospital of Eastern Ontario
	401 Smyth Road
	Ottawa, Ontario
	K1H 8 L1
	Phone: 613-737-7600 ext. 2538
	Email: menon@cheo.on.ca
Secondary sponsor	Children’s Hospital of Eastern Ontario Research Institute
Contact for public queries	KM, Pediatric Critical Care, Children’s Hospital of Eastern Ontario, Ottawa, Canada
Contact for scientific queries	KM, Pediatric Critical Care, Children’s Hospital of Eastern Ontario, Ottawa, Canada
Public title	Steroids in Fluid and Vasoactive Infusion Dependent Shock (STRIPES) pilot study
Scientific title	Steroids in Fluid and Vasoactive Infusion Dependent Shock (STRIPES) pilot study
Country of recruitment	Canada, multi- academic center (7) study
Health problem under investigation	Efficacy and safety of hydrocortisone as a treatment of fluid and vasoactive infusion dependent shock
Key inclusion and exclusion criteria	Eligible for study: started on a vasoactive infusion within 24 h of PICU admission
	Inclusion criteria: newborn to 17 years of age, receiving vasoactive infusions for 1–6 h
	Exclusion criteria: known or suspected hypothalamic, pituitary or adrenal disease; currently receiving steroids for shock prior to randomization; are expected to have treatment withdrawn; post cardiac surgery; primary cardiogenic shock, spinal shock, hemorrhagic, or hypovolemic shock proven or strongly suspected; previously enrolled in the STRIPES study; steroids started for reasons other than shock; no longer on inotropes at the time of randomization or first dose of study drug; or physician refusal
Study type	Pragmatic, multi-center, double-blind, pilot randomized controlled trial
Date of first enrollment	September 4, 2014
Target sample size	72
Recruitment status	Recruiting as of July 2014
Primary outcome	Patient accrual rate over a 1-year recruitment period
Key secondary outcomes	Adherence to the study protocol; frequency of open-label corticosteroid use and the clinical characteristic of patients in whom open-label corticosteroids are used; incidence of mortality and adverse events; time to discontinuation of inotropes; and the feasibility of mechanistic blood sampling

### Study setting

The STRIPES pilot study will recruit patients from the emergency department (ED), pediatric ward, and/or pediatric intensive care unit (PICU) of seven academic pediatric centers in Canada (a list of participating study sites is available on ClinicalTrials.gov).

### Patient enrollment

**Fig. 1:**
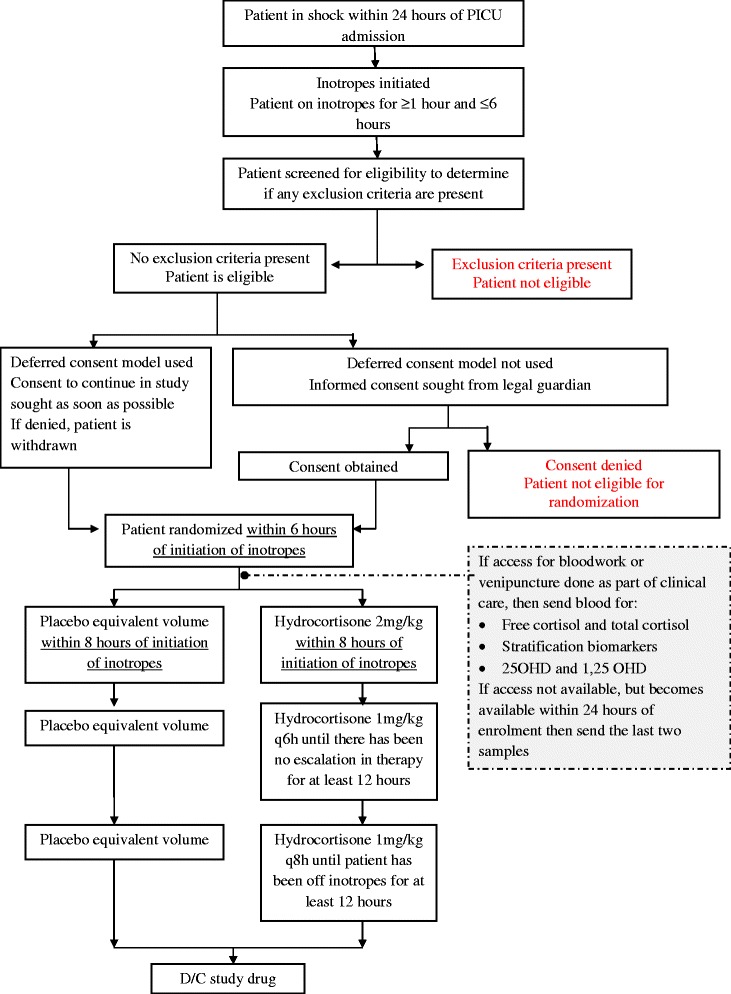
Protocol flow diagram for the STRIPES pilot study

ICU-based clinical research assistants will screen pediatric ICU patients for eligibility criteria which include being a patient with fluid and vasoactive infusion dependent shock who has received inotropic support for at least 1 h, and no more than 6 h, within their first 24 h of PICU admission. This short period for recruitment is necessary as shock is a progressive process characterized by an *early compensated phase* in which adaptive mechanisms maintain blood pressure and tissue perfusion, an *uncompensated phase* where these mechanisms fail but the patient may still respond to therapeutic interventions, and a final *irreversible stage* where shock progresses to permanent organ and tissue injury and even death [29]. It is critical that any potential therapy, including corticosteroids, be provided early, which is why we have chosen a 6-h cut-off for enrollment. The rationale for this time window therefore is to enroll patients early in shock when steroids may have the potential to prevent or reverse end-organ injury, prior to the stage of advanced or irreversible shock. The detailed inclusion and exclusion criteria are summarized in Table 2. Eligible patients will be identified in the ED and PICU by nurses, attending physicians, and trainees at participating sites at the time the patient is started on a vasoactive agent (see Fig. 1 for the protocol flow diagram).

**Table 2:** STRIPES pilot study inclusion and exclusion criteria

Inclusion criteria	
1. Children newborn to 17 years and on any dose of any vasoactive infusion for between 1 to 6 h	
Exclusion criteria	
1. Known or suspected hypothalamic, pituitary, or adrenal disease	
2. Currently receiving steroids for shock prior to randomization	
3. Expected to have life support withdrawn	
4. Premature infants (<38 weeks corrected gestational age)	
5. Pregnancy	
6. Post cardiac surgery	
7. First dose of vasoactive infusion administered >24 h after PICU admission	
8. No longer on vasoactive infusion at time of enrollment and/or expected to no longer be on vasoactive infusion at time first dose of study drug due	
9. Primary cardiogenic shock suspected or proven	
10. Spinal shock suspected or proven	
11. Hemorrhagic or hypovolemic shock suspected or proven	
12. Previously enrolled in STRIPES	
13. Started on vasoactive infusion for reasons not related to shock	
14. Physician refusal

### Consent

The initial resuscitation of a pediatric patient is a highly stressful situation during which parents are often unable to contemplate involvement in a research trial and/or may not be available within the time frame required by a given study. Therefore, as per the Canadian Tri-Council guidelines [30], we will be seeking deferred consent through the Research Ethics Board (REB) applications. Informed consent will be obtained from the legal guardian of all patients; however, when the deferred consent model is employed, informed consent will be obtained following enrollment. Using this model, children will be enrolled and randomized when they are determined to be eligible for the study. A member of the local study team will approach the legal guardian for written informed consent to continue study participation as soon as possible after enrollment. If consent is obtained, the patient will remain in the study. If consent is denied, the legal guardian and patient will be provided the option of having patient data and the blood sample destroyed. In centers where a deferred consent model is not approved by the REB, informed consent will be sought from the legal guardian in writing or by telephone, after which the patient will be enrolled and randomized. Legal guardians may provide consent for data collection and drug administration with or without blood sampling.

### Randomization

Randomization will employ a password-protected, web-based system. Patients will be randomized in a 1:1 ratio using random variable block sizes (2–4 patients/block), and randomization will be stratified by site to account for site-specific practice variation. The randomization system will assign each randomized patient a unique study ID number. The site pharmacist will match the ID number to a treatment arm on a hard-copy randomization list and dispense the appropriate treatment. At sites where a research pharmacist is not available on evenings and weekends, numbered medication kits will be accessible to research assistants in a temperature-controlled refrigerator.

### Allocation concealment and blinding

All study personnel (the overall study research coordinator, research assistants, site investigators, Principal Investigator, co-investigators, data management personnel, and statisticians), members of the health care team (treating physicians, bedside nurses, and clinical pharmacists if different from the research pharmacist), and patients/families will be blinded to the study group assignment. To maintain blinding, the randomization lists will only be accessible to the Methods Centre at the Ottawa Hospital Research Institute and to the site pharmacies. The active drug and the placebo (hydrocortisone and normal saline) will be identical in appearance, volume, and smell as hydrocortisone is made up in normal saline and dissolves completely with no visible precipitate.

Study procedures are in place to address the possibility of requests by treating physicians to unblind group allocation for a particular patient. The treating physician will notify the site investigator, who will ask the pharmacy to reveal to which group the patient has been allocated. We expect that the request to unblind patients will be minimal as treating physicians will be permitted to use open-label corticosteroids if they judge that this is clinically necessary.

### Interventions

Patients randomized to the hydrocortisone group will receive a 2-mg/kg hydrocortisone intravenous (IV) bolus at the time of enrollment followed by 1 mg/kg of hydrocortisone administered intravenously every 6 h (q6h) until the patient has not had an escalation in therapy (as defined by an increase in their vasoactive infusions or a fluid bolus such as normal saline, Ringer’s lactate, albumin, or any other blood product) for at least 12 h. Once these criteria are met, hydrocortisone will be reduced to 1 mg/kg every 8 h and continue at this frequency until all vasoactive infusions have been discontinued for 12 h. The weaning of hydrocortisone from q6h to q8h is important in order to ensure the patient receives the minimum amount of corticosteroid that is necessary for hemodynamic stability so as to prevent adrenal suppression and potential adverse events. If, following the initial hydrocortisone wean, the patient requires fluid boluses and/or an increase in their vasoactive infusion(s), the dosing frequency will return to every 6 h until the patient meets stability criteria again. Hydrocortisone will be continued for a maximum of 7 days to minimize the incidence of adrenal suppression. In keeping with the pragmatic nature of this trial, all other patient management, including (but not limited to) use of intubation, mechanical ventilation, sedation and analgesia, hemodynamic triggers and endpoints, red cell transfusions, antibiotics, and fluid boluses, will be left to the discretion of the treating physician. The Surviving Sepsis Campaign guidelines flowchart (see [31]) will be attached to the study protocol for easy reference by the treating physician, but its use will not be mandated; however, the use of vasoactive infusions and other therapies will be recorded.

Although we will discourage physicians from using open-label corticosteroids and carefully record any such occurrence, we will not refer to open-label use as a protocol violation so as not to deter enrollment and to encourage buy-in. In the event that the patient does not receive the full treatment as per protocol, data collection will continue.

Patients in the control group will receive a placebo consisting of normal saline equivalent in volume to the appropriate dose of hydrocortisone. The remainder of the protocol will be as per the experimental group.

### Blood sampling

Although the primary focus of this pilot study is to determine the feasibility of conducting a clinical outcome-based RCT of hydrocortisone versus placebo in shock, this pilot also provides an excellent opportunity to perform some exploratory mechanistic studies. Given the expertise of our team, the specific substudies we chose to conduct include the utility of free cortisol versus total cortisol measurements, the ability of stratification biomarkers to predict severity of illness, and the interaction between the adrenal and vitamin D axes. More detail on these substudies is provided in Additional file 2. Patients with existing venous or arterial access and who are undergoing a venipuncture for clinical blood work before the first dose of study drug will have a blood sample sent for analysis of free cortisol and total cortisol, mortality risk stratification biomarkers, 25-hydroxyvitamin D, and 1,25-hydroxyvitamin D. If access for bloodwork is not available before the study drug is initiated, but it becomes available within the first 24 h of enrollment, a research sample will be sent for analysis of stratification biomarkers, 25-hydroxyvitamin D, and 1,25-dihydroxyvitamin D only.

### Outcomes

This is a pilot study; hence, the primary outcome is feasibility, defined by accrual rate over 1 year. Our goal is to recruit 72 patients within this time frame. However, we will consider patient accrual to be adequate if we recruit 60 patients from seven sites within 1 year. We will also assess potential barriers to recruitment, including lack of a deferred consent model, physician- and guardian-related consent issues, availability of research personnel, and the narrow recruitment window (6 h from the initiation of vasoactive infusion). The secondary outcomes are summarized in Table 3; they include the frequency of open-label steroid use, adverse events related to corticosteroid use, time to discontinuation of vasoactive infusions, incidence of mortality, and the feasibility of blood sampling.

**Table 3:** Secondary outcomes for the STRIPES pilot study

Outcome	Metric for analysis
Adherence to the protocol:	We will consider adherence adequate if each of goals a through c is met in 80 % of enrolled patients:
a. Time to administration of the first dose of study drug b. Weaning of drug to q8h when hemodynamically stable c. Discontinuation of drug when off all vasoactive infusions	a. Goal is <8 h from starting vasoactive medication b. Goal is to wean within 12 h of no escalation of therapy c. Goal is to discontinue between 12 and 18 h after vasoactive infusion stopped
Open-label steroid use	We will consider the number of patients started on open-label steroids to be acceptable if it occurs in <10 % of patients
Clinical outcomes:	
a. Adverse events b. Clinical endpoints	a. Severe bleeding, secondary infections, and use of insulin infusions b. Time to discontinuation of vasoactive infusion and incidence of mortality
Successful blood sampling and processing	The percentage of patients from whom blood samples are sent
	The percentage of samples sent that are successfully received and analyzed in their respective labs

An RCT design is necessary to investigate our feasibility outcomes. The main goal of our study is to determine the feasibility of conducting an RCT of hydrocortisone versus placebo in pediatric septic shock as determined by our ability to recruit patients into this trial. There were several issues with potential recruitment that we identified a priori that could only be tested through a pilot RCT. Firstly, we identified that some centers could have issues with recruiting patients into such a trial because they wished to administer steroids. Thus, determining whether or not they will actually randomize patients is of paramount importance. Secondly, the study has a very narrow recruitment window; therefore, we need to determine whether or not sites can randomize patients, obtain the study drug, and administer the first dose within 8 h of patient eligibility. This can only be tested by piloting the randomization procedures. Finally, we are proposing a novel deferred consent model for obtaining informed consent. It will be very important to understand the acceptability of this model for families in the context of a randomized double-blind controlled trial.

A randomized controlled trial design is also necessary to determine if physicians will administer open-label steroids to patients if they worsen. It would be important to determine if the use of open-label steroids would be different between the two groups: Does early administration of steroids lead to physiologic improvements that remove the necessity for open-label steroids?

### Sample size

Based on data from our multi-center retrospective study, we expect to enroll 72 patients. This target takes into account the frequency of septic shock at each center, along with the projected consent rate at each center. This target number will: (1) allow us to assess our feasibility objectives over a reasonable time period (1 year), (2) allow each center to recruit between 6 and 24 patients, and (3) allow us to test the acceptability of our eligibility criteria as well as open-label steroid use at seven sites and with exposure to 50 different clinicians. Given that this is a pilot study, we will consider recruitment to be feasible if we achieve 80 % (60 patients) of our target enrollment, a threshold commonly used in critical care pilot studies. With 60 patients, we will have the ability to detect an adherence rate of 80 ± 10 % (meaning 80 ± 10 % of study patients will have fewer than 10 % of monitored values as violations). The median time to discontinuation of vasoactive agents observed in this pilot study will be used to better estimate the sample size needed for the full RCT.

### Recruitment, compliance, and follow-up

All ED and PICU staff will be made aware of the study through information sessions, posters in the ED and PICU, weekly rounds with the research assistants, and weekly emails. Monthly recruitment newsletters will be sent to all sites to encourage enrollment and discuss commonly encountered questions.

Compliance is likely to be high as the protocol is simple and closely follows usual clinical practice in these patients. This assumption is based on experience from the Vasopressin in Pediatric Shock (VIP) study, which randomized children in vasodilatory shock to vasopressin or placebo and used many of the same sites and investigators as our current proposal. Despite utilizing a more complex protocol and recruiting sicker patients, the VIP trial had only two protocol violations and was successfully completed [32]. It is possible that open-label corticosteroid use will be higher than the anticipated 10 % rate. However, we will collect specific data on the circumstances in which this occurs in order to provide more targeted education around this practice in the full trial. Follow-up for the STRIPES pilot study ends at hospital discharge or death. Therefore, we anticipate a follow-up rate of 100 %.

### Data collection and management

Data for each patient will be entered by trained research assistants at each site and managed using an electronic data capture tool, REDCap (Research Electronic Data Capture), which will be hosted at the Children’s Hospital of Eastern Ontario (CHEO) Clinical Research Unit [33]. REDCap is a secure, web-based application designed to support data collection for research studies. Predefined ranges for all data values will be set up in this application to allow data entry personnel to validate data as soon as it is entered and send data queries immediately. Missing data will be similarly managed. For each enrolled patient, we will collect information from their medical chart. Data will be entered into the case report form (CRF, available on the STRIPES study website at http://stripes.ccctg.ca/Home.aspx), and each record will be identified only by study ID number to maintain patient confidentiality. The following information will be collected: demographic information, inotrope use, duration of mechanical ventilation, PRISM III and PELOD-2 scores, insulin infusion use, gastrointestinal bleeding, laboratory results, and basic resource utilization data. Research assistants at each site will maintain a daily paper screening log which will track every patient who is started on a vasoactive infusion within their first 24 h of PICU admission. When eligible patients are not enrolled, we will classify the reason for non-enrollment as: (1) refusal from patient and/or legal guardian (specifying reason for refusal, if provided); (2) inability to contact legal guardian in centers without deferred consent (specifying reason contact could not be made, if available); (3) refusal from attending physician (specifying reason for refusal); (4) lack of availability of the research assistant; and (5) patient died before enrollment. We will also record age, gender, and PRISM III scores for these patients to determine if there are demographic differences between eligible patients who are and are not enrolled. A data management plan has been developed for the study and is available on the STRIPES study website.

### Statistical analysis

To meet the feasibility objectives of this pilot RCT, we have planned descriptive analyses. We will present recruitment rate, feasibility events, and open-label corticosteroid use as proportions with 95 % confidence intervals. Recruitment feasibility will be defined as achieving 80 % of our target goal of 72 patients. We will present continuous data as means and standard deviations, or medians and interquartile ranges, as appropriate. Patients who are randomized but withdraw or do not provide consent will be analyzed using intention to treat if they provided consent to keep their data.

### Monitoring

The Data Monitoring and Safety Committee (DMSC) will include a senior biostatistician, a pediatric endocrinologist, and a pediatric intensive care specialist. The DMSC will review all serious adverse events (SAEs) — any serious events that the attending site intensivist believes may be directly related to enrollment in this trial. Study sites will report all SAEs to the Coordinating Center within 24 h of becoming aware of the event. The site will fill out a SAE report and provide any related clinical documentation (de-identified) to the Coordinating Center for distribution to the Principal Investigator and the DMSC chair. The DMSC chair will determine whether immediate input from other DMSC members is required before sending the final DMSC determination to the Principal Investigator. There will be no stopping rules; however, the DMSC can make recommendations to the Principal Investigator, who will communicate back to the Steering Committee at the end of the trial regarding any safety concerns for the full trial. We will provide the DMSC with analyses by group (blinded as group A and B) at the completion of this pilot RCT. These analyses will include relative rates of gastrointestinal bleeding, infections, and hospital mortality. Due to the small sample size and short duration of this pilot trial, we have not planned for any interim analyses.

The study coordinator will monitor the sites at study initiation, midway through the recruitment period, and at study close-out to ensure compliance with the protocol and to troubleshoot any barriers to recruitment. Data entered into the electronic CRF will be verified on an ongoing basis, and the Data Quality function in REDCap will be used to send and resolve data queries where required. Given the pilot nature of this study, a formal audit of the trial will not be conducted.

### Ethics

Research ethics board approval has been obtained from all participating centers. Protocol amendments will be communicated as necessary to those involved with the study. An application for deferred consent was approved at five sites (Ottawa, Vancouver, Calgary, Hamilton, and Halifax). Deferred consent was not possible in Quebec as per provincial legislation. The study protocol was approved by the following ethics review boards: the Children’s Hospital of Eastern Ontario Research Ethics Board, Ottawa, Ontario (reference number: 14/05E); the Hamilton Integrated Research Ethics Board, Hamilton, Ontario (reference number: 14–636); Comité d’éthique de la recherche à CHU Sainte-Justine, Montreal, Quebec (reference number: 3990); the Montreal Children’s Hospital Research Ethics Board, Montreal, Quebec (reference number: 14-121-PED); the Conjoint Health Research Ethics Board of the University of Calgary, Calgary, Alberta (reference number: REB14-0606); the University of British Columbia Children’s and Women’s Research Ethics Board, Vancouver, British Columbia (reference number: H14-01109); and the IWK Research Ethics Board in Halifax, Nova Scotia (reference number: 1017182).

### Close-out

All data and source documentation will be stored in a locked, secure storage facility for 25 years from the time of study close-out. The study drug will be destroyed and reconciled at each site according to the procedures of the site pharmacy. Only the Principal Investigator, the research coordinator, or their delegate will have access to the data once the study is closed.

## Discussion

We recently published a systematic review [6] on the use of corticosteroids in pediatric shock. The review concluded that “The literature on the use of steroids in pediatric shock is limited in amount, methodological quality and demonstrates conflicting results. The limited evidence on which current guidelines are based strongly supports the need for a well-designed, pragmatic randomized controlled trial on the use of steroids in pediatric shock to inform future guidelines.” There are two small RCTs currently listed on www.clinicaltrials.gov (NCT01047670, NCT00732277); however, neither study has been updated since 2010. Given their size and unclear recruitment status, it is unlikely that either of the latter two trials will be able to answer the question of whether corticosteroids improve outcomes in pediatric shock.

This study is the first step towards a large RCT to provide clarity on the use of corticosteroids in critically ill children with shock. Results of the STRIPES pilot study will provide essential feasibility data for planning and conducting a larger, multi-center trial that will help to establish the role of corticosteroids in children with fluid and vasoactive infusion dependent shock. Our goal is to enroll a minimum of 60 patients at seven sites over a 1-year period. Failure to do so will prompt us to modify our plans for a future trial. If our recruitment rate is as anticipated or better, we will not modify eligibility criteria. If our recruitment rate is marginal (that is, barely achieves our goals), we will examine the number of patients excluded on the basis of each exclusion criterion, and will reconsider the necessity for any criterion that has resulted in a large number of excluded patients. We will record the number of eligible non-randomized patients and reasons for non-enrollment, and on the basis of these results, we will consider deterrents to randomization and methods to enhance enrollment of eligible patients. If the pilot study demonstrates feasibility, no major protocol changes are needed, and no safety concerns are raised by the STRIPES DMSC, then the results of the pilot study will be rolled into the full trial. However, if any of the above criteria are not met, then the protocol will be re-evaluated and the feasibility results of the pilot study published independently.

### Trial status

Recruitment for the STRIPES pilot study started at the Coordinating Centre (Children's Hospital of Eastern Ontario, Ottawa) in July 2014. Four of the six other Canadian PICUs initiated recruitment between December 2014 and February 2015. The remaining two sites will commence recruitment in July of 2015 and in the fall of 2015, respectively. The first patient was enrolled on September 4, 2014, and recruitment is expected to continue until March of 2016.

## Additional files

Additional file 1: SPIRIT Checklist. (DOC 143 kb) Additional file 2: Summary of mechanistic studies. This table summarizes the mechanistic studies that will be performed as part of the STRIPES pilot study, including the rationale, timing, and amount of blood sample required for each test. (PDF 202 kb)

## Abbreviations

DMSC: Data Monitoring and Safety Committee
ED: emergency department
IV: intravenous
PELOD: pediatric logistic organ dysfunction
PICU: pediatric intensive care unit
PRISM: pediatric risk of mortality
RCT: randomized controlled trial
REB: Research Ethics Board
STRIPES: Steroid Use in Fluid and Vasoactive Infusion Dependent Shock
VIP: Vasopressin in Pediatric Shock
