# Supplementary material for: Steroids in fluid and/or vasoactive infusion dependent pediatric shock: study protocol for a randomized controlled trial
Source: Trials. 2016 May 6;17:238. doi: 10.1186/s13063-016-1365-6 (PMC4859989; doi:10.1186/s13063-016-1365-6)
Supplement: Additional file 2: — Summary of mechanistic studies. This table summarizes the mechanistic studies that will be performed as part of the STRIPES pilot study, including the rationale, timing, and amount of blood sample required for each test. (PDF 202 kb) [file 13063_2016_1365_MOESM2_ESM.pdf]

Additional file 2: Summary of Mechanistic Studies

Analysis	Rationale	Timing of Test	Amount of Blood Required
Free cortisol versus total cortisol	The ideal method for measurement of adrenal function in critical illness is highly controversial. Adrenocorticotropic hormone stimulation testing is expensive, cumbersome and difficult to interpret [1, 2] random total cortisol levels may be affected by serum protein levels in critical illness [3-5] and have not consistently correlated with clinically important outcomes [1, 6]. Some recent studies have suggested that free cortisol levels may better reflect adrenal function in critical illness but these studies have not been linked to outcomes in an RCT [7, 8].	Prior to administration of study drug	0.6 ml
Stratification biomarkers	It is currently difficult to predict the outcome of patients with septic shock. Previous studies have shown that younger age[9] was a risk factor for illness severity in these patients. However, more recent gene-expression based work [10, 11] has supported the existence of septic shock sub-classes that predict mortality based on a 100-gene expression signature. Further validation of this finding as a stratification tool for clinical trials is still needed.	Within 24 hours of admission	0.1 ml
25 hydroxyvitamin D and 1,25 hydroxyvitamin D	The hemodynamic status of a patient in shock is a complex interplay between a variety of autonomic, endocrine and physiologic parameters. Although cortisol is felt to play an important role in this hemodynamic status, a recent study by our group suggested that Vitamin D deficiency may have an additive effect on hemodynamic instability [12].	Within 24 hours of admission	0.5 ml

1. Crowley S, Hindmarsh PC, Honour JW, Brook CG. Reproducibility of the cortisol response to stimulation with a low dose of ACTH(1-24): the effect of basal cortisol levels and comparison of low-dose with high-dose secretory dynamics. J Endocrinol. 1993;136(1):167-72.
2. Nye EJ, Grice JE, Hockings GI, Strakosch CR, Crosbie GV, Walters MM et al. Adrenocorticotropin stimulation tests in patients with hypothalamic-pituitary disease: low dose, standard high dose and 8-h infusion tests. Clin Endocrinol (Oxf). 2001;55(5):625-33.
3. Beishuizen A, Thijs LG, Vermes I. Patterns of corticosteroid-binding globulin and the free cortisol index during septic shock and multitrauma. Intensive care medicine. 2001;27(10):1584-91. doi:10.1007/s001340101073.

4. Hamrahian AH, Oseni TS, Arafah BM. Measurements of serum free cortisol in critically ill patients. The New England journal of medicine. 2004;350(16):1629-38. doi:10.1056/NEJMoa020266.
5. Zimmerman JJ DS, Barker RM, et al. Real-time cortisol quantification among critically ill children. Pediatr Crit Care Med. 2010.
6. Hatherill M, Tibby SM, Hilliard T, Turner C, Murdoch IA. Adrenal insufficiency in septic shock. Arch Dis Child. 1999;80(1):51-5.
7. Poomthavorn P, Lertbunrian R, Preutthipan A, Sriphrapradang A, Khlairit P, Mahachoklertwattana P. Serum free cortisol index, free cortisol, and total cortisol in critically ill children. Intensive care medicine. 2009;35(7):1281-5. doi:10.1007/s00134-009-1480-x.
8. Bendel S, Karlsson S, Pettila V, Loisa P, Varpula M, Ruokonen E et al. Free cortisol in sepsis and septic shock. Anesth Analg. 2008;106(6):1813-9. doi:10.1213/ane.0b013e318172fdba.
9. Menon K, Ward RE, Lawson ML, Gaboury I, Hutchison JS, Hebert PC et al. A prospective multicenter study of adrenal function in critically ill children. Am J Respir Crit Care Med. 2010;182(2):246-51. doi:10.1164/rccm.200911-1738OC.
10. Wong HR, Salisbury S, Xiao Q, Cvijanovich NZ, Hall M, Allen GL et al. The pediatric sepsis biomarker risk model. Crit Care. 2012;16(5):R174. doi:10.1186/cc11652.
11. Wong HR, Weiss SL, Giuliano JS, Jr., Wainwright MS, Cvijanovich NZ, Thomas NJ et al. Testing the prognostic accuracy of the updated pediatric sepsis biomarker risk model. PLoS One. 2014;9(1):e86242. doi:10.1371/journal.pone.0086242.
12. McNally JD, Menon K, Chakraborty P, Fisher L, Williams KA, Al-Dirbashi OY et al. The association of vitamin D status with pediatric critical illness. Pediatrics. 2012;130(3):429-36. doi:10.1542/peds.2011-3059.
